# Should Reinke edema be considered a contributing factor to post-extubation failure?

**DOI:** 10.1186/s13054-015-1147-7

**Published:** 2015-12-19

**Authors:** Andrea Cortegiani, Vincenzo Russotto, Santi Maurizio Raineri, Antonino Giarratano

**Affiliations:** Department of Biopathology and Medical Biotechnologies (DIBIMED), Section of Anesthesia, Analgesia, Intensive Care and Emergency, Policlinico P. Giaccone, University of Palermo, Via del Vespro 129, 90127 Palermo, Italy

We read with interest the recently published review in *Critical Care* about post-extubation laryngeal edema and stridor by Pluijms et al. [1]. The review considers in detail the risk factors for post-extubation respiratory failure and describes a post-extubation algorithm for its prevention and reduction.

We recently published a case report describing the occurrence of post-extubation stridor leading to post-extubation respiratory failure in a woman with a previously undiagnosed Reinke edema (RE) [2]. RE is a progressive laryngeal soft-tissue swelling. The condition typically manifests in female gender as hoarseness and as a gradually deepening voice in patients with a history of smoking, vocal cord abuse, and/or gastroesophageal reflux. Other case reports of RE complicating airway management already exist [3]. Our patient did not undergo prolonged intubation or difficult airway instrumentation. However, she presented risk factors for RE (i.e. female gender and smoking history). Notably, female gender has been reported in different studies investigating risk factors for complications following extubation which have been summarized in the review by Pluijms et al. [1].

Since our patient did not present stridor before intubation, the additional laryngeal edema due to airway management, which would have not been responsible for post-extubation failure in normal conditions, contributed to reaching the critical obstruction of airways. We believe, in accordance with other authors [4], that RE may be considered an important, underdiagnosed concomitant cause of post-extubation stridor. RE may explain why, in certain patients, a minor grade of laryngeal edema could lead to a clinically relevant reduction of airway space and post-extubation stridor. Clinical signs and risk factors for RE should be systematically assessed when clinicians deal with risks of post-extubation failure. In our opinion, when risk factors for RE are associated with difficult airway management and/or prolonged intubation, a conservative approach consisting of otolaryngology consultation, fiberoptic examination, or a neck computed tomography scan may be undertaken for a safe airway assessment and detection of a potentially critical obstruction.

## Abbreviation

RE: Reinke edema
